# Process Optimization and Validation of Functional Pasta Reformulated with Lupin and Flaxseed Flours: Impacts on Technological Quality, Bioactive Potential, and Sensory Attributes

**DOI:** 10.1007/s11130-026-01557-2

**Published:** 2026-08-10

**Authors:** Luz María Paucar-Menacho, Alicia Lavado-Cruz, Rebeca Salvador-Reyes, Anggie Verona-Ruiz, John Gonzales-Capcha, Soledad Quezada-Berrú, Cesar Moreno-Rojo, Sander Moreira Rodrigues, Marcio Schmiele

**Affiliations:** 1 Departamento Académico de Agroindustria y Agronomía, Facultad de Ingeniería, Universidad Nacional del Santa, Nuevo Chimbote, 02712 Peru; 2 Facultad de Ingeniería, Universidad Tecnológica del Perú, Lima, 150101 Peru; 3 Laboratório Integrado de Cereais e Lipídeos (LICEL), Instituto de Ciência e Tecnologia (ICT), Universidade Federal dos Vales do Jequitinhonha e Mucuri (UFVJM), Diamantina, 39100-000 Brazil

**Keywords:** *Lupinus mutabilis*, Tarwi, *Linum usitatissimum*, Response surface methodology, Functional pasta, Antioxidant capacity, Technological quality

## Abstract

Traditional wheat-based pasta is widely consumed; however, its reformulation with functional plant-based ingredients remains challenging because technological quality, bioactive properties, and consumer acceptance must be balanced. This study aimed to optimize and validate a functional pasta formulation by partially replacing wheat flour (WF) with lupin flour (LF) and flaxseed flour (FF). A 2² central composite rotatable design was applied, using LF (2–12%) and FF (5–15%) as independent variables. Instrumental color, cooking properties, texture, total soluble phenolic compounds (TSPC), antioxidant capacity (ORAC), and sensory acceptance were evaluated. Flour substitution significantly affected pasta quality, particularly color, cooking behavior, and texture. Increasing FF reduced lightness and increased redness, whereas higher LF contributed to yellowness. Although cooking and texture properties were modified, several formulations maintained acceptable technological performance, with low cooking loss and suitable texture-related attributes. Enriched pasta showed significantly higher TSPC and ORAC values than the control, indicating enhanced bioactive potential. Sensory evaluation revealed moderate to high acceptance, although excessive substitution negatively affected color and texture perception. Sensory acceptance was mainly associated with texture, stickiness, and cooking time. Numerical optimization identified an optimal formulation with 12.0% LF and 12.13% FF, achieving an overall desirability of 77.3%. Validation assays showed good agreement between predicted and experimental values, confirming model adequacy. Overall, lupin and flaxseed flours are promising ingredients for developing functional pasta with improved bioactive potential and acceptable technological and sensory quality.

**Supplementary Information:**

The online version contains supplementary material available at https://doi.org/10.1007/s11130-026-01557-2.

## Introduction

 Pasta is one of the most widely consumed foods worldwide due to its versatility, affordability, and ease of preparation. Traditional wheat-based pasta remains dominant because its gluten network forms a viscoelastic matrix that provides firmness, low solid loss, and structural integrity during cooking [1]. However, conventional pasta is primarily carbohydrate-based and contains low levels of protein, dietary fiber, and bioactive compounds, promoting its reformulation with ingredients of greater functional value [2]. In this context, pasta has gained attention as a suitable matrix for incorporating functional ingredients, agro-industrial by-products, and alternative protein and fiber sources [2], although enrichment may also affect color, texture, cooking time, and sensory acceptability [2, 3].

Legumes are particularly relevant for pasta reformulation because they provide protein, fiber, vitamins, minerals, and essential amino acids that are less abundant in wheat, such as lysine and threonine [1, 4]. Among them, *tarwi* or Andean lupin (*Lupinus mutabilis*) stands out for its high protein content, relevant lipid fraction, mineral composition, and absence of gluten, making it an attractive raw material for nutritionally enhanced foods. Nevertheless, its industrial use remains limited, mainly to whole grains and flour, which highlights an opportunity for value addition [5]. Lupin has also shown promising performance in plant-based formulations by improving protein content and amino acid profile [6].

Flaxseed (*Linum usitatissimum*) is another promising ingredient due to its high content of polyunsaturated fatty acids, dietary fiber, lignans, and phenolic compounds. It is also considered a strategic crop, with an oil content close to 40% and a lipid profile of interest for functional food development [7]. In food applications, flaxseed has been associated with improvements in omega-3 fatty acid content, dietary fiber, functional performance, and antioxidant properties. For example, plant-based formulations combining flaxseed with lupin and soy showed improved protein profile and stable physicochemical characteristics [6], while fermented flaxseed-based products exhibited favorable functional and antioxidant properties, although sensory optimization remains necessary.

Nevertheless, pasta enrichment should not focus exclusively on nutritional composition. Acceptable technological properties must also be preserved, since pasta quality depends on water absorption, solid loss, firmness, elasticity, stickiness, and color, which influence sensory acceptance and industrial feasibility [1, 2]. Recent studies have confirmed that plant-derived ingredients, fiber-rich by-products, and alternative protein sources can improve pasta nutritional quality but may also modify its technological behavior, making formulation optimization and validation essential before industrial application [3].

Within this framework, combining lupin and flaxseed flours represents a promising strategy, as lupin contributes high-quality protein and minerals, whereas flaxseed provides dietary fiber and functional lipids, especially omega-3 fatty acids [5]. However, limited evidence is available on how this combination affects the relationship between functional attributes, technological quality, and sensory acceptance in enriched pasta. Therefore, this study aimed to optimize and validate a functional pasta formulation incorporating lupin and flaxseed flours, providing scientific support for the development of value-added pasta with improved nutritional and bioactive potential without compromising technological and sensory quality.

## Materials and Methods

The detailed methodology is provided in the Supplementary Material (Supplementary Methodology).

## Results and Discussion

### Nutritional and Physical Properties of the Raw Materials

The chemical composition, antioxidant-related properties, instrumental color, and particle size distribution of WF, FF, and LF are presented in Table S1 (Supplementary Material). Compared with WF, both LF and FF exhibited a more favorable nutritional profile, characterized by a significantly lower digestible carbohydrate content and higher levels of total dietary fiber, protein, lipids, and ash. These results confirm the potential of both flours as functional ingredients to enhance the compositional profile of pasta formulations through higher protein and dietary fiber contents.

Among the three raw materials, LF showed the highest protein (38.55 ± 0.45 g/100 g d.w.) and total dietary fiber contents (32.01 ± 0.83 g/100 g d.w.), whereas FF exhibited the highest lipid content (35.29 ± 0.03 g/100 g d.w.). The proximate composition of FF was generally consistent with previous reports for whole flaxseed flour, which typically contains 35.2–43.2% lipids, 20.2–21.0% protein, and 2.0–2.6% ash [8]. Similarly, the high protein content observed in LF agrees with the values reported for lupin flour by Plustea et al. [9], who described it as a protein-rich ingredient containing approximately 30.95% protein, 12.42% lipids, 3.67% ash, and 45.8% carbohydrates. The higher protein and dietary fiber contents found in the present study may be attributed to differences in lupin species, growing conditions, and analytical methodologies. Conversely, WF presented the highest digestible carbohydrate content (75.66 ± 0.69 g/100 g d.w.) and lower concentrations of protein, dietary fiber, ash, and lipids. These differences highlight the nutritional complementarity of LF and FF as ingredients for wheat flour fortification, particularly due to their higher protein, dietary fiber, and lipid contents compared with WF.

Regarding antioxidant-related parameters, flaxseed flour (FF) exhibited the highest total soluble phenolic compound content (195.80 ± 11.03 mg GAE/g d.w.), followed by lupin flour (LF) (129.44 ± 10.47 mg GAE/g d.w.), whereas wheat flour (WF) showed the lowest value (55.40 ± 5.56 mg GAE/g d.w.). The elevated phenolic content of FF is mainly attributed to its abundance of lignans, particularly secoisolariciresinol diglucoside (SDG), together with phenolic acids and flavonoids, which contribute substantially to its antioxidant properties [10]. Although LF presented a lower TSPC than FF, it showed the highest ORAC value (122.68 ± 19.54 µmol TE/g d.w.), suggesting that the antioxidant activity may also be influenced by the specific composition and reactivity of individual bioactive compounds [11, 12]. These findings indicate that both flaxseed and lupin flours can enhance the functional and antioxidant potential of pasta formulations, with flaxseed contributing mainly through phenolic enrichment and lupin through a combination of phenolic compounds and other antioxidant constituents.

Instrumental color analysis revealed marked differences among the raw materials. WF showed the highest lightness value (L* = 89.56 ± 0.22), together with low a* and b* values, indicating a typical whitish appearance, consistent with the characteristics reported for wheat flour by Yaver and Bilgiçli [13]. LF showed lower lightness (L* = 74.89 ± 0.04) and the highest b* value (24.40 ± 0.52), indicating a more yellowish color. In contrast, FF exhibited the lowest L* value (64.99 ± 0.43) and the highest positive a* value (4.26 ± 0.04), reflecting the darker and more reddish-brown appearance associated with the seed coat.

Particle size distribution also differed among flours. WF showed a more heterogeneous distribution, with 85.84% of particles between 125 and 250 μm. In contrast, LF exhibited a coarser profile, with 90.71% of particles between 250 and 600 μm, while FF showed 81.10% of particles within the same range. Similar trends have been reported for lupin and flaxseed flours, which generally exhibit larger particle sizes than wheat flour due to their higher protein, fiber, and lipid contents [8, 9]. These differences may affect water absorption, dough development, and mixing behavior during processing because particle size influences the specific surface area available for hydration and the physical interactions among flour components. Coarser particles generally hydrate more slowly and less uniformly than finer particles, leading to heterogeneous dough development and reduced continuity of the gluten network. In addition, the coarse fiber-rich particles of lupin and flaxseed may physically disrupt the gluten matrix and modify water distribution within the dough, thereby influencing mixing behavior, extrusion performance, cooking quality, and the textural properties of the final pasta product [4, 14].

While Andean lupin and flaxseed naturally contain antinutritional compounds, including quinolizidine alkaloids, cyanogenic glycosides, and phytates, the concentrations of these constituents are expected to be markedly reduced by the processing steps employed in the present study. The lupin seeds were subjected to a debittering treatment prior to flour production, a process widely recognized for its effectiveness in lowering alkaloid content to levels considered safe for human consumption. In addition, unit operations such as drying, milling, pasta processing, and cooking have been reported to further reduce the concentration, bioavailability, and biological activity of antinutritional factors. Consequently, these processing conditions likely contributed to improving the nutritional quality and safety of the final product. Nevertheless, the direct quantification of antinutritional compounds was not performed and therefore represents a limitation of the present study. Future investigations should include the determination of residual alkaloids, cyanogenic glycosides, and phytates to provide a more comprehensive assessment of the nutritional and safety attributes of the developed pasta formulations.

### Validation of the Rheological Properties of Wheat Flour

The validation of the rheological properties of wheat flour is presented in Supplementary Material (Validation of the Rheological Properties of Wheat Flour).

### Effect of Wheat Flour Replacement With Lupin and Flaxseed Flours on Pasta Quality

**Fig. 1:**
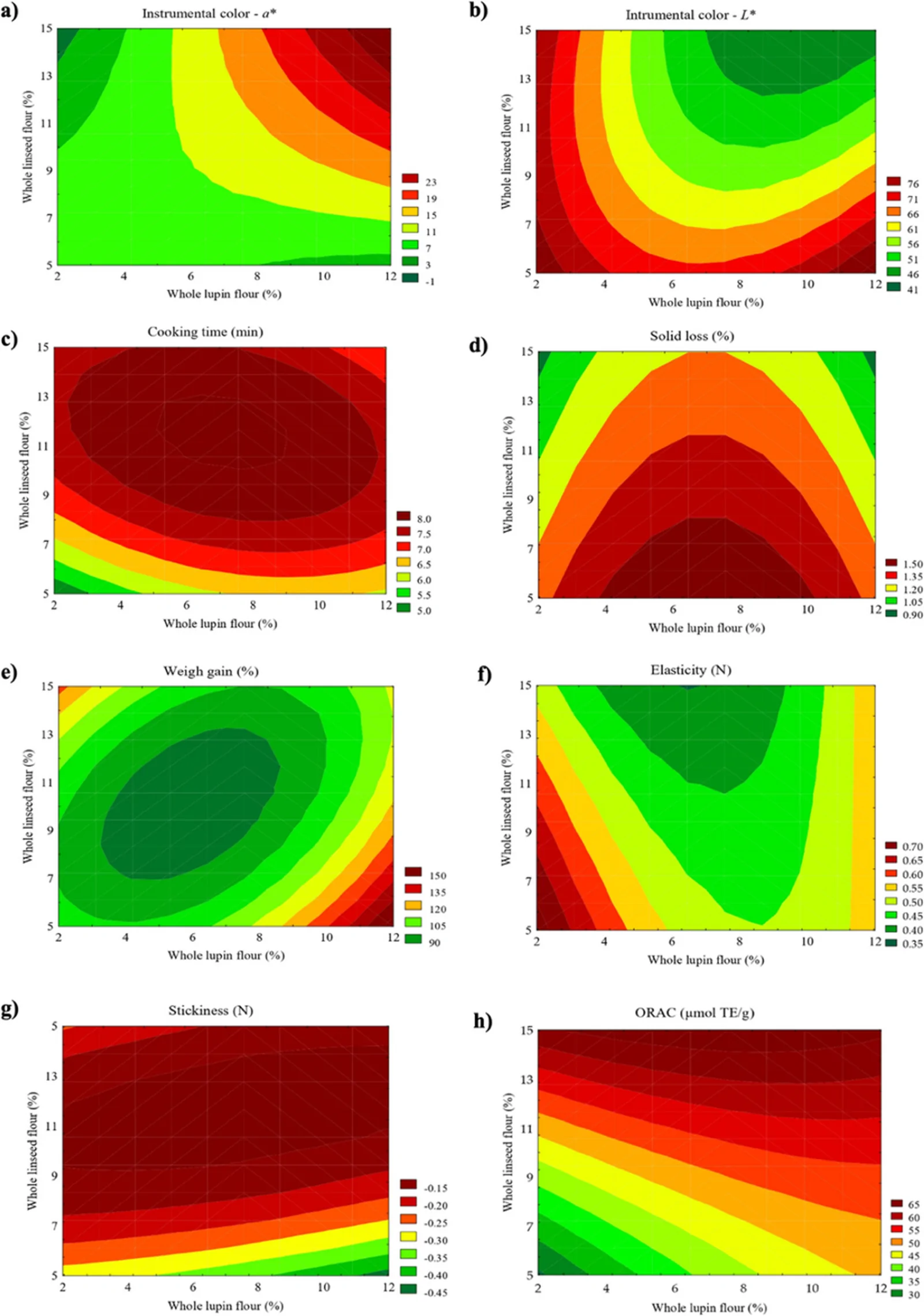
Contour plots showing the effect of lupin flour (%) and flaxseed flour (%) on pasta quality parameters: (**a**–**b**) color parameters (L* and a*); (**c**–**e**) cooking properties (cooking time, solid loss, and weight gain); (**f**–**g**) texture properties (elasticity and stickiness) and (**h**) antioxidant capacity

The effects of wheat flour replacement with LF and FF on pasta quality were assessed in terms of technological performance, antioxidant-related properties, and sensory acceptance. Detailed results for instrumental color, cooking properties, texture parameters, total soluble phenolic compounds, antioxidant capacity, and sensory scores are provided in Supplementary Tables S2–S4. Representative images of the pasta formulations are presented in Supplementary Figure S1. The predictive regression models selected for response surface analysis and optimization are shown in Table 1, and the corresponding contour plots are presented in Fig. 1.

**Table 1:** Predictive regression models for potential technological properties and bioactive potential of pasta (*p* < 0.05 and adjusted R² > 0.80).

Dependent variable	Mathematical model	*p*-value	*R* ^2^ (predicted)	*R* ^2^ (adjusted)
L*	$$\:\begin{array}{c}y=51.28-5.31x_1+\:7.18x_1^2\\-7.67x_2-\:2.19x_2^2-4.60x_1x_2\end{array}$$	0.001	94.29	88.59
a*	$$\begin{array}{c}\:y=6.85+5.59x_1+\:0.44x_1^2\\+3.59x_2-0.61x_2^2+\:5.55x_1x_2\end{array}$$	0.011	92.59	85.18
Cooking time (min)	$$\begin{array}{c}\:y=7.99+0.13x_1-\:0.37x_1^2\\-\:0.55x_2-\:0.62x_2^2-0.25x_1x_2\end{array}$$	0.000	84.66	69.32
Weight gain	$$\begin{array}{c}\:y=94.05+10.24x_1+16.42x_1^2\\-4.49x_2+11.45x_2^2-12.75x_1x_2\end{array}$$	0.021	97.92	95.82
Solid loss (%)	$$\begin{array}{c}\:y=1.36+0.002x_1-\:0.12x_1^2\\-0.11x_2-0.01x_2^2-0.02x_1x_2\end{array}$$	0.002	86.29	72.58
Elasticity (N)	$$\begin{array}{c}\:y=0.41-0.02x_1+\:0.08x_1^2\\-0.04x_2-0.03x_1x_2\end{array}$$	0.007	80.27	62.54
Stickiness (N·s)	$$\begin{array}{c}\:y=-0.19-0.01x_1-\:0.01x_1^2\\+0.07x_2-0.07x_2^2+0.03x_1x_2\end{array}$$	0.001	90.73	81.47
ORAC (µmol TE/g d.w.)	$$\begin{array}{c}\:y=51.35+2.58x_1-\:0.96x_1^2\\+6.66x_2+0.59x_2^2-1.56x_1x_2\end{array}$$	0.001	84.17	68.34

Abbreviations: x_1_ Lupin flour; x_2_ Flaxseed flour; ORAC, oxygen radical absorbance capacity; TE, Trolox equivalent

### Technological Quality

#### Instrumental Color

Pasta color is a key quality attribute because it strongly influences consumer perception and overall product acceptability. In the present study, the lightness (L*) values of the pasta formulations ranged from 41.59 ± 0.03 to 72.23 ± 0.05, whereas the redness parameter (a*) ranged from 0.17 ± 0.03 to 22.48 ± 0.02 (Supplementary Table S2). Both responses were adequately described by the fitted regression models (Table 1), which showed high predictive capacity for L* (R² = 94.29%) and a* (R² = 92.59%). Increasing the levels of LF and FF significantly decreased L* values, resulting in darker pasta samples. This effect was more pronounced for FF, which showed the strongest negative linear effect on lightness. In addition, the interaction between both ingredients further contributed to the reduction in L*, indicating that simultaneous substitution intensified pasta darkening. This behavior is consistent with previous reports attributing decreases in noodle lightness to the brown pigments and seed coat components naturally present in flaxseed flour [15].

A similar trend was observed for the a* parameter. Both flours exerted significant positive linear effects on redness, and their interaction also contributed positively to this response, indicating a shift toward redder tones as substitution levels increased. These results agree with previous studies reporting darker and redder pasta products after flaxseed incorporation [16]. The contour plots shown in Fig. 1a-b confirm these trends, showing that higher FF levels decreased L* and increased a*, producing darker and redder pasta samples. In contrast, the yellowness parameter (b*) ranged from 10.12 ± 0.05 to 41.99 ± 0.07, but this response was not satisfactorily explained by the fitted model and was therefore not considered in the response surface analysis. Nevertheless, all formulations showed positive b* values, indicating a yellow coloration that became more evident at higher LF levels. This effect may be associated with the natural yellow pigments present in lupin, as previously observed in lupin-enriched pasta products [17].

#### Cooking Properties

Cooking time ranged from 6 to 8 min (Table S3 in Supplementary Material), and the predictive model explained 84.66% of the response variability (Table 1; Fig. 1c). Both LF and FF significantly affected the time required to reach the optimal cooking point, indicating that flour substitution modified the hydration dynamics and structural organization of the pasta matrix. Weight gain during cooking was mainly influenced by the quadratic effects of both independent variables, and the model showed strong predictive performance (R² = 97.92%), indicating that intermediate-to-high substitution levels favored greater water absorption. Cooking loss ranged from 1.04 to 1.49%, values that were slightly higher than those of the control but still well below the threshold generally considered acceptable for high-quality pasta (< 6%). The fitted model also showed adequate predictive capacity for this response (R² = 86.29%).

The observed changes in cooking behavior can be explained by the distinct composition and hydration properties of the substituted flours. Flaxseed flour contains mucilage rich in soluble non-starch polysaccharides, which can increase viscosity, limit water mobility, and delay starch gelatinization, thereby influencing cooking time and water absorption [18, 19]. In parallel, lupin proteins exhibit high water-binding capacity and may interact with the starch–protein matrix, contributing to structural stability and reduced solid loss during cooking despite partial gluten dilution [20]. Similar behavior has been reported in pasta enriched with lupin flour and other legume flours, where moderate substitution levels maintained low cooking losses and satisfactory technological performance [21, 22]. Therefore, the low cooking loss values observed in the present study suggest that the combined action of lupin proteins and flaxseed mucilage helped preserve the integrity of the pasta matrix during cooking.

Likewise, pasta formulations enriched with chickpea and lentil flours exhibited low cooking losses and satisfactory technological performance at moderate substitution levels. The authors attributed this behavior to legume proteins’ ability to interact with the starch–protein matrix, thereby contributing to structural stability during cooking despite partial dilution of gluten. These findings support the results obtained in the present study, suggesting that the incorporation of protein-rich plant ingredients can enhance the nutritional quality of pasta while maintaining acceptable cooking performance and product integrity [22]. Furthermore, previous reviews have highlighted that the water-binding properties of flaxseed proteins and soluble fibers, particularly mucilage polysaccharides, can enhance hydration behavior and contribute to the stabilization of cereal-based matrices during cooking, supporting the results obtained in the present study [19].

#### Instrumental Texture

Fracturability ranged from 1.06 to 2.56 N, firmness from 1.73 to 2.68 N, and work of shear from 1.04 to 1.80 N, with most enriched formulations showing lower values than the control. However, these responses were not retained for response surface discussion because their models showed limited predictive performance. In contrast, elasticity and stickiness were adequately described by the fitted models, with R² values of 80.27 and 90.73%, respectively. The quadratic effect of LF had the main influence on elasticity, whereas the interaction between LF and FF significantly affected stickiness. The contour plots in Fig. 1f-g illustrate the combined influence of both flours on these responses.

Overall, the texture results suggest that partial replacement of wheat flour modified the structure of the pasta matrix through a balance between gluten dilution and the structuring effects of lupin proteins and flaxseed mucilage. The generally lower fracturability, firmness, and shear work can be attributed to the weakening of the continuous gluten network. In contrast, the behavior of elasticity and stickiness indicates that soluble fibers and protein-fiber interactions played a relevant role in modulating the technological quality of the enriched pasta [13, 23].

The textural properties of the pasta formulations were strongly influenced by the interactions among wheat gluten, lupin proteins, and flaxseed polysaccharides. The partial replacement of wheat flour with lupin flour resulted in gluten dilution, which may have reduced the continuity and strength of the gluten network, thereby affecting firmness and fracturability. Nevertheless, the high protein content of lupin flour likely contributed to the formation of a cohesive protein–starch matrix capable of partially compensating for the reduction in gluten functionality and preserving structural integrity during processing and cooking. Furthermore, flaxseed flour contains soluble mucilage polysaccharides with a high water-binding capacity, which can modify dough hydration, alter water distribution within the matrix, and influence the viscoelastic properties of the final product. Similar effects have been reported in pasta enriched with fiber-rich ingredients, where moderate incorporation levels maintained acceptable firmness and technological quality despite partial disruption of the gluten network. Likewise, Pérez-Lozano et al. [24] demonstrated that the incorporation of legume proteins in combination with hydrocolloids enhanced pasta structure through protein–polysaccharide interactions, resulting in improved cohesiveness and texture stability. Therefore, the texture profile observed in the present study appears to be the result of a balance between gluten dilution and the structuring effects provided by lupin proteins and flaxseed mucilage, which contributed to maintaining acceptable technological quality in the enriched pasta.

### Total Soluble Phenolic Compounds and Antioxidant Capacity

#### Total Soluble Phenolic Compounds 

TSPC content of the pasta formulations ranged from 272.70 ± 10.31 to 348.99 ± 5.62 mg GAE/100 g d.w., whereas the control sample showed a substantially lower value (121.03 ± 1.94 mg GAE/100 g d.w.). The highest TSPC content was observed in formulation F3, while the lowest value among the enriched samples corresponded to F10. These results indicate that the incorporation of LF and FF markedly increased the phenolic content of pasta (Supplementary Table S3).

Although the regression analysis indicated significant effects for some model terms, the overall model showed only moderate explanatory power (R² = 0.77) together with a significant lack of fit (*p* < 0.05). Therefore, this response was not considered suitable for response surface analysis or optimization. The increase in TSPC observed in the enriched formulations can be attributed to the natural phenolic compounds contributed by both raw materials, particularly flaxseed, which is known to contain lignans and phenolic acids such as secoisolariciresinol diglucoside, matairesinol, and pinoresinol [25]. Similar trends have been reported in previous studies on flaxseed-enriched noodles and pasta products [15, 26].

#### Antioxidant Capacity

Antioxidant capacity, determined by the ORAC assay, ranged from 45.30 ± 0.44 to 63.12 ± 1.11 µmol TE/g d.w. in the enriched formulations, showing a marked increase compared with the control sample (15.46 ± 1.11 µmol TE/g d.w.). The fitted model adequately explained the variability of the data (R² = 84.17%), allowing this response to be included in the response surface analysis and optimization model (Table 1). Both LF and FF exerted significant positive linear effects on ORAC values, whereas their interaction had a negative effect.

The contour plot shown in Fig. 1h indicated that the highest ORAC values were obtained at higher FF levels combined with intermediate-to-high LF levels. This behavior was evident in formulation F4, which showed the highest antioxidant capacity (63.12 ± 1.11 µmol TE/g d.w.), representing an approximately 4.1-fold increase relative to the control. These results highlight the important contribution of flour substitution to enhancing the antioxidant potential of pasta formulations.

Similar findings have been reported in previous studies. Duan et al [27] observed that the incorporation of flaxseed meal into dried Chinese noodles increased antioxidant activity, although it also affected product color. Likewise, Madenci et al. [28] reported that ingredients rich in dietary fiber and antioxidant compounds improve the functional properties of pasta. Other studies on legume- and seed-enriched pasta have also shown increases in phenolic compounds and antioxidant capacity, even after processing or digestion [29]. In the present study, the higher ORAC values observed in enriched samples suggest that antioxidant capacity was influenced not only by phenolic compounds but also by other bioactive constituents and possible synergistic interactions between the ingredients.

### Sensory Acceptance and Purchase Intention

Sensory analysis was conducted to assess the sensory acceptance and purchase intention of enriched fresh pasta formulations among semi-trained panelists. In general, pasta containing LF and FF showed a slight reduction in acceptance compared with the control pasta prepared with 100% WF. Nevertheless, the sensory scores remained within an acceptable range, indicating moderate to high consumer acceptance across the enriched formulations. Furthermore, no bitterness, astringency, or other undesirable sensory attributes commonly associated with residual alkaloids were reported by the sensory panelists during product evaluation. This finding provides indirect evidence that the debittering process was effective in reducing alkaloid levels to concentrations that did not negatively affect the sensory quality of the pasta. Although alkaloid quantification was not performed, the absence of adverse sensory perceptions related to bitterness supports the suitability of the debittered lupin flour for use in pasta production.

The partial replacement of WF with LF and FF influenced the evaluated sensory attributes, including color, flavor, texture, and appearance. Overall acceptance scores ranged from 6.30 ± 1.26 to 7.60 ± 1.53, suggesting that the incorporation of both flours did not drastically compromise product acceptability. As expected, the control sample showed the highest scores for all evaluated attributes. However, some enriched formulations, particularly F7 and F5, showed values close to those of the control, indicating that appropriate substitution levels can preserve satisfactory sensory quality.

Color was one of the attributes most visibly affected by flour substitution. Formulations with higher substitution levels, such as F3 and F8, showed lower color acceptance scores than the control, suggesting that the darker and redder tones associated with flaxseed flour negatively influenced visual perception. This trend agrees with previous reports showing that incorporation of legume or seed flours may intensify pasta color and reduce consumer preference when substitution levels are high [30]. In contrast, moderate substitution levels may improve the compositional profile of pasta by increasing protein and dietary fiber contents without substantially affecting color acceptability [16].

Texture also played an important role in consumer response. Although enriched pasta may develop a firmer structure that contributes to an “al dente” character, excessive substitution can reduce elasticity and increase stickiness, thereby negatively affecting sensory perception. In the present study, this behavior was more evident in formulations with higher flaxseed flour content, such as F4, which showed lower texture and overall acceptance scores. Conversely, formulations with intermediate substitution levels tended to show more favorable sensory profiles, suggesting a better balance between technological quality and consumer perception.

Overall, the sensory results indicate that moderate levels of wheat flour replacement with LF and FF can produce enriched pasta with acceptable consumer perception, whereas higher substitution levels may reduce acceptability due to less favorable changes in color, texture, flavor, and appearance. These findings are consistent with previous studies reporting that intermediate substitution levels provide the best compromise between sensory quality and nutritional improvement [31].

### Relationship Between Sensory and Technological Properties

Pearson correlation analysis showed that purchase intention was strongly associated with the acceptance index (*r* = 0.98). Instrumental firmness and fracturability correlated positively with sensory texture (*r* = 0.61 and 0.64), whereas stickiness and cooking time correlated negatively with texture, flavor, and acceptance (*r* = −0.70 to −0.88). Higher FF content also reduced acceptance and sensory texture (*r* = −0.65 and − 0.66). Detailed results and scatter plots are provided in Figs. S2–S16 of the Supplementary Material.

### Numerical Optimization and Model Validation

Numerical optimization was performed using the statistically significant dependent variables with acceptable model fit (*P* < 0.10; R² > 0.80). Cooking time, elasticity, stickiness, and ORAC were set to be maximized, whereas L* and solid loss were set to be minimized. The a* parameter was maintained within the desired range (Fig. S17 in Supplementary Material). The optimal formulation corresponded to 12.0% LF and 12.13% FF, equivalent to coded levels of 1.41 and 0.60, respectively, with an overall desirability of 77.3%.

The optimized formulation was prepared in true triplicate, and the experimental values obtained were 51.93 ± 0.46 for L*, 7.01 ± 0.09 for a*, 7.63 ± 0.13 min for cooking time, 144.69 ± 3.79% for weight gain, 1.10 ± 0.08% for solid loss, 0.58 ± 0.03 N for elasticity, − 0.16 ± 0.02 N·s for stickiness, and 52.63 ± 3.52 µmol TE/g d.w. for ORAC.

The relative deviations between predicted and experimental values were lower than 10% for all responses, indicating good agreement between model predictions and validation results. These findings confirm the adequacy of the fitted models for predicting pasta quality responses and support the validity of the optimization strategy applied in this study.

Considering the optimized formulation, the estimated nutritional composition on a dry basis was 19.55% protein, 8.35% lipids, 1.28% ash, 13.11% total dietary fiber, and 57.68% digestible carbohydrates. Based on this composition, an 80 g serving of dry pasta would provide approximately 15.6 g of protein and 10.5 g of dietary fiber, corresponding to 28–31 and 38–42% of the recommended daily intakes for healthy adults, respectively [32, 33]. Compared with conventional commercial wheat pasta marketed in Peru, which generally provides approximately 12 g of protein and 3 g of dietary fiber per 100 g, the optimized formulation showed a clearly improved nutritional profile. Moreover, its protein content was comparable to that reported for commercial quinoa–tarwi pasta products, while its dietary fiber content was higher than the declared values for these products. Overall, these results support the potential of lupin and flaxseed flours to obtain a value-added pasta with enhanced protein and dietary fiber contents.

## Conclusions

Partial replacement of wheat flour with lupin flour (LF) and flaxseed flour (FF) significantly influenced the technological quality, bioactive potential, and sensory attributes of pasta. Both ingredients improved the functional profile, particularly by increasing total soluble phenolic compounds and antioxidant capacity, while also modifying color and texture. LF mainly increased yellowness, whereas FF promoted darkening, redness, and antioxidant capacity. The fitted regression models adequately described the principal responses retained for optimization, including color, cooking properties, texture-related parameters, and ORAC. Despite partial dilution of the gluten matrix, several formulations maintained acceptable cooking and textural performance, particularly at intermediate substitution levels. Sensory evaluation showed moderate-to-high acceptance, although excessive substitution reduced preference because of less favorable color and texture. Correlation analysis further supported the relationship between sensory perception and texture, stickiness, and cooking time.

Numerical optimization identified an optimal formulation containing 12.0% LF and 12.13% FF, with an overall desirability of 77.3% and good agreement between predicted and experimental values. Overall, combining lupin and flaxseed flours represents a promising strategy for producing functional pasta with enhanced bioactive potential while maintaining acceptable technological and sensory quality.

## Supplementary Information

Below is the link to the electronic supplementary material.

Supplementary Material 1 (DOCX 7.06 MB)

## Data Availability

No datasets were generated or analysed during the current study.
