# Supplementary material for: Process Optimization and Validation of Functional Pasta Reformulated with Lupin and Flaxseed Flours: Impacts on Technological Quality, Bioactive Potential, and Sensory Attributes
Source: Plant Foods Hum Nutr. 2026 Aug 10;81(3):106. doi: 10.1007/s11130-026-01557-2 (PMC13457343; doi:10.1007/s11130-026-01557-2)
Supplement: Supplementary file 1 [file 11130_2026_1557_MOESM1_ESM.docx]

**Process Optimization and Validation of Functional Pasta Reformulated with Lupin and Flaxseed Flours: Impacts on Technological Quality, Bioactive Potential, and Sensory Attributes**

Plant Foods for Human Nutrition

Luz María Paucar-Menacho, Alicia Lavado-Cruz, Rebeca Salvador-Reyes, Anggie Verona-Ruiz, John Gonzales-Capcha, Soledad Quezada-Berru, Cesar Moreno-Rojo, Sander Moreira Rodrigues, and Marcio Schmiele

Affiliations: Universidad Nacional del Santa, Perú; Universidad Tecnológica del Perú, Perú; Universidade Federal dos Vales do Jequitinhonha e Mucuri, Brasil

Corresponding authors: luzpaucar@uns.edu.pe; marcio.sc@ict.ufvjm.edu.br

**Supplementary Material**

**Supplementary Methodology**

**Raw Materials**

The raw materials used were durum wheat flour (*Triticum durum*, commercially refined wheat flour; Nicolini, Peru), commercially debittered Andean lupin seeds (*Lupinus mutabilis* Sweet, variety Altagracia) from Ancash, Peru, brown flaxseed *(Linum usitatissimum*, variety Curahuasi), drinkable water and commercial whole *in natura* eggs purchased at the local market. The use of commercially dehulled lupin seeds ensured a substantial reduction in quinolizidine alkaloids before flour production.

**Preparation of Flours**

Commercially debittered Andean lupin (*Lupinus mutabilis* S.) seeds were selected, cleaned, and manually peeled to remove the seed coat. The debittered seeds were then dehydrated in a tray dryer for 12 h at 60 °C. The dried material was milled in a hammer mill (TORRH, Jarcon del Perú SRL, Junín, Perú) and sieved through a 0.1 mm mesh opening to obtain a particle size between 150 and 200 µm. The resulting flour was vacuum-packed (Besser Vacuum, Auster, Udine, Italia) and stored under refrigeration at 8 °C. Flaxseed was previously washed, selected, and manually sorted to remove impurities. Moisture content was then reduced by drying in a tray dryer at 50 °C for 24 h. The dried material was milled in a hammer mill (TORRH, Jarcon del Perú SRL, Junín, Per) and sieved through a 0.1 mm mesh opening to obtain a particle size between 150 and 200 µm. The flour was vacuum-packed (Besser Vacuum, Auster, Udine, Italia) and stored under refrigeration at 8 °C.

**Proximate Composition and Physical Properties of Raw Materials**

The proximate composition of the raw materials, including moisture, protein, fat, and ash was determined according to AACCI (American Association of Cereal Chemists International) approved methods 44-15.02, 46-13.01, 30-10.01, and 08-03.01, respectively [1]. Total dietary fiber (TDF) was determined using the K-RINTDF assay kit and expressed on a dry weight basis. Particle size distribution was evaluated according to the AOAC method 965-22 [2]. Digestible carbohydrate content was calculated by difference. All compositional results are expressed on a dry weight basis (d.w.). In addition, the rheological properties of the wheat flour were characterized using a farinograph (Brabender, SQ 810161, Germany) and extensograph (Brabender, 860723, Germany according to AACCI methods and 54-21.02 and 54-10.01, respectively [1]. Instrumental color was measured using the CIELab system [3].

**Experimental Design and Pasta Formulation**

Pasta formulation was optimized using a 2² central composite rotational design, generated with Design-Expert software (version 7, Stat-Ease Inc., Minneapolis, MN, USA), comprising four factorial points, four axial points, and three central points, for a total of 11 experimental runs. The independent variables were the proportions of lupin flour (LF) and flaxseed flour (FF) used to partially replace wheat flour (WF). The coded and actual values of the independent variables are shown below.

**Table.** Central composite rotatable design (CCRD 2²) used for pasta formulation optimization

| Formulation | Point type | Coded values | | Actual values | |
| --- | --- | --- | --- | --- | --- |
|  |  | X_1_ | X_2_ | LF | FF |
| F1 | Factorial | -1 | -1 | 3.5 | 6.5 |
| F2 | Factorial | 1 | -1 | 10.5 | 6.5 |
| F3 | Factorial | -1 | 1 | 3.5 | 13.5 |
| F4 | Factorial | 1 | 1 | 10.5 | 13.5 |
| F5 | Axial | -α | 0 | 2 | 10.0 |
| F6 | Axial | +α | 0 | 12 | 10.00 |
| F7 | Axial | 0 | -α | 7 | 5.0 |
| F8 | Axial | 0 | +α | 7 | 15.0 |
| F9 | Central | 0 | 0 | 7 | 10.0 |
| F10 | Central | 0 | 0 | 7 | 10.0 |
| F11 | Central | 0 | 0 | 7 | 10.0 |

Abbreviations: X_1_, lupin flour (LF); X_2_, flaxseed flour (FF).

In addition, a control formulation prepared with 100% wheat flour was included for comparative purposes. For each treatment, 1000 g of flour blend was mixed with 250 mL of whole *in natura* egg, and 100 mL of water and processed in a Pastaia 2 pasta extruder (Italvisa Máquinas LTDA, Brazil) equipped with a fettuccine die, with a 2 kg hopper capacity, 0.46 kVA power, and a production capacity of 5–9 kg/h. The resulting pasta strands (approximately 28 cm) were dried at 24 °C for 24 h, packaged, and stored at 4 °C until analysis.

**Technological Properties of Pasta**

***Instrumental Color***

The color of raw functional pasta samples was evaluated using the CIELab tristimulus color system with a Color 3° colorimeter [3]. The parameters measured were lightness, chroma, and hue angle. The instrument was configured with illuminant D65, a 10° observation angle, and RSIN calibration mode.

***Optimal Cooking Time***

A 20 g pasta sample was cooked in 280 mL of boiling distilled water. Optimal cooking time was determined through the AACCI method 66-50.01[1] by compressing the pasta strand between two glass slides every 30 s until the central white core disappeared, indicating complete starch gelatinization.

***Weight Gain***

Samples were weighed before and after cooking at their optimal cooking time. Weight gain was evaluated by the method 66-50.01 form AACCI [1] and expressed as the percentage ratio between cooked pasta weight and raw pasta weight.

***Cooking Loss***

Cooking loss was determined according to the method 66-50.01 from AACCI [1] by evaporating 25 mL of the cooking water obtained during the weight gain analysis and drying the residue in an oven at 105 °C until constant weight was reached.

***Instrumental Texture***

The texture properties of the pasta were evaluated using a ta TAXT Plus texture analyzer (Micro Stable System, Haslemere, UK). The fracturability of the pasta was determined using a 3-Point Bending Rig probe (HDP/3PB). Ten replicate measurements were conducted on single fettuccine-type pasta strands, following the approach described by Silva et al. [4]. Test conditions included a test speed of 0.50 mm·s^−1^, a height of 20 mm, a distance of 5 mm in compression mode, and a force threshold of 0.049 N. For firmness and work of shear determination, 30 g of pasta samples were cut into 5 cm strands and cooked in 420 mL of distilled water according to AACCI method 16-50.01 [1]. After cooking, ten replicate measurements were conducted on six pasta strands for each repetition. A A/LKB-F probe was used with pre-test and test speeds of 0.50 mm·s^−1^, a post-test speed of 10.0 mm·s^−1^, a height of 15 mm, a distance of 14 mm, and a force threshold of 0.049 N.

Pasta elasticity was determined using 30 g of fresh pasta strands of 28 cm length after cooking for the time previously established in the cooking test in 420 mL of distilled water. After draining, tensile force was applied to the pasta strand until rupture, with ten replications. The A/SPR probe test parameters included a test speed of 3.0 mm·s^−1^ and a distance of 70 mm [7].

**Phenolic Compounds and Antioxidant Capacity**

***Total Soluble Phenolic Compounds***

The total soluble phenolic compound content of flour and pasta samples was determined using the Folin–Ciocalteu method following Pico et al. [5], with minor modifications. Briefly, 100 mg of the sample was extracted with 1 mL of 80% methanol acidified with 0.1% hydrochloric acid. Samples were Thermomixer (Eppendorf AG, Hamburg, Germany) and centrifuged (Centrifuge 5424 R, Eppendorf AG, Hamburg, Germany) at 5,000 RCF for 15 min. The supernatant was collected, while the residue was re-extracted with 1 mL of 70% acetone in 0.1% hydrochloric acid under the same conditions. Both extracts were combined and adjusted to a final volume of 2 mL with distilled water. Then, 1 mL of extract was mixed with 100 µL of Folin reagent diluted to 0.1% in distilled water and 100 µL of 5% NaOH. After incubation for 120 min at room temperature in the dark, absorbance was measured at 750 nm using a Synergy H1 microplate reader (BioTek Instruments, USA). Results were calculated using a gallic acid standard curve (0–225 µg·mL^−1^) and expressed as mg gallic acid equivalents (GAE) per 100 g dry weight.

***Oxygen Radical Absorbance Capacity***

Antioxidant activity was determined using the oxygen radical absorbance capacity (ORAC) assay according to Paucar-Menacho et al. [6]. Briefly, reaction mixtures were prepared with 30 µL of sample or Trolox standard in 75 mM phosphate buffer (pH 7.4), 180 µL of 70 nM fluorescein, and 90 µL of 12 mM 2,2′-azobis(2-amidinopropane) dihydrochloride. The mixtures were placed in black 96-well microplates, and fluorescence was measured every 2 min for 2.5 h at excitation and emission wavelengths of 485 and 520 nm, respectively, using a Synergy H1 microplate reader (BioTek Instruments, USA). Results were expressed as µmol Trolox equivalents (TE) per g dry weight based on a Trolox standard curve ranging from 0 to 160 µM (R² ≥ 0.99).

**Sensory Analysis**

Sensory tests were conducted in accordance with ethical guidelines approved by the Universidad Nacional del Santa (PIC. FF. II. 006-2025). Panelists read and signed an Informed Consent Form (ICF) and analyzed the samples in individual booths with white light in the Sensory Analysis Laboratory of the Agroindustrial Pilot Plant. To minimize sensory fatigue and carry-over effects, the eleven pasta formulations of the experimental design and the control sample were not evaluated simultaneously. Instead, the sensory analysis was conducted in three independent evaluation sessions, with the treatments randomly distributed among sessions. Session 1 included treatments 1, 5, 8, and 11; Session 2 included treatments 3, 6, 7, and 9; and Session 3 included treatments 2, 4, 10, and control. Each panelist evaluated only the samples assigned to the corresponding session under identical testing conditions. The order of sample presentation within each session was randomized, and samples were identified using three-digit random codes.

Sensory evaluation was conducted with 30 semi-trained panelists (male and female, aged 18–45 years) who had been previously familiarized with sensory evaluation procedures for food products. The samples were evaluated using a hedonic scale to assess their sensory attributes. This experimental design was intended to provide a comparative assessment of the different formulations rather than to represent a large-scale consumer acceptance test. Similar studies on legume-enriched noodle products have used semi-trained panels of 30 individuals familiar with sensory evaluation procedures, including prior training in the use of hedonic and bipolar scales to ensure consistency in scoring [7]. Furthermore, the number of participants required for sensory acceptability testing depends on the study objectives and variability, rather than on a fixed sample size [8].

Pasta samples were cooked to their optimal cooking time, coded with random three-digit numbers, and presented monadically in random order. Panelists evaluated color, flavor, texture, and appearance using a 9-point hedonic scale, where 1 corresponded to “dislike extremely” and 9 to “like extremely”. Purchase intention was also assessed using a 5-point hedonic scale, where 1 corresponded to “certainly would not buy” and 5 to “certainly would buy”. In a sensory session, the panelists were presented with the 11 coded samples in random order, and to minimize carryover effects between samples, panelists rinsed their mouths with drinkable water.

**Statistical Analysis**

All experimental results were expressed as mean ± standard deviation. The normality of the data was checked using the Shapiro–Wilk test. Response surface methodology (RSM) was applied to evaluate the effects of the independent variables on the technological, functional and sensory characteristics of pasta formulations using a 7.0 Statistica software (Statsoft Inc., Tulsa, OK, USA). For numerical optimization, only statistically significant models (P < 0.10) with adequate fit (R² ≥ 0.80) were considered. Optimization was carried out using the desirability function approach based on the significant dependent variables obtained from the regression models using Design-Expert 13 software (Stat-Ease Inc., Minneapolis, MN, USA) [9]. The optimal formulation was then produced and analyzed in triplicate.

**Validation of the Rheological Properties of Wheat Flour**

The farinographic properties of wheat flour indicated a water absorption capacity of 60.7% and a dough consistency of 600 FU. Dough development time was 5.32 min, stability was 18.65 min, and the mixing tolerance index was 24 FU. The relatively high stability value indicates good dough strength and tolerance to mixing, which is desirable for pasta processing. Extensographic analysis performed after 30, 60, and 90 min showed resistance values of 1257, 1638, and 1638 EU, respectively. The corresponding farinographic profile is shown in the following figure.

**Figure S1.** Farinograph profile of wheat flour illustrating dough development time (DDT), stability (S1), mixing tolerance index (MTI), and consistency parameters during mixing.

Overall, these results indicate that the wheat flour exhibited rheological characteristics consistent with a strong flour suitable for fresh pasta production. Parameters such as dough development time, stability, and resistance to extension suggest the formation of a sufficiently strong gluten network, which is essential for maintaining dough structure during processing. Strong flours are typically associated with higher protein quality, greater water absorption capacity, and improved viscoelastic performance [10, 11]. The flour also showed good extensibility after 30 min of resting, confirming its suitability for producing fresh pasta with adequate mechanical and technological quality [12]. This characteristic was particularly important in the present study because wheat flour was partially replaced with non-gluten flours (lupin and flaxseed), which can weaken the gluten network. Therefore, the use of a strong wheat flour helped preserve dough structure and processing performance during extrusion.

**Correlation Analysis**

Pearson correlation analysis was used to examine relationships between sensory perception and technological characteristics. The scatter plots are provided in Figs. S3–S16. A strong positive association was observed between the acceptance index and purchase intention (r = 0.98). Sensory texture was positively correlated with instrumental firmness (r = 0.61) and fracturability (r = 0.64), showing agreement between instrumental and sensory texture measurements.

Stickiness was negatively correlated with sensory texture (r = -0.81), flavor (r = -0.71), and the global sensory score (r = -0.70). Cooking time was also negatively correlated with sensory texture (r = -0.88) and the global sensory score (r = -0.80), indicating that formulations requiring longer cooking times were less well accepted.

Flaxseed flour content was negatively correlated with the global sensory score (r = -0.65) and sensory texture (r = -0.66). These findings indicate that sensory acceptance depended not only on nutritional enhancement but also on maintaining appropriate texture, stickiness, and cooking behavior.

**References**

1. AACC International (2010) Approved methods of analysis, 11th edn. Methods 08-03.01, 30-10.01, 44-15.02, 46-13.01, 54-10.01, 54-21.02, and 66-50.01. AACC International, St. Paul, MN

2. AOAC International (1966) AOAC Official Method 965.22: Sorting corn grits. Sieving method. First Action 1965; Final Action 1966. AOAC International, Rockville, MD. <https://www.aoacofficialmethod.org/index.php?main_page=product_info&cPath=1&products_id=1846>

3. Konica Minolta Sensing, Inc. (2007) Precise color communication: Color control from perception to instrumentation. Konica Minolta Sensing, Inc. <https://www.konicaminolta.com/instruments/knowledge/color/pdf/color_communication.pdf>

4. Silva LEPD, Moreira SR, Neves NDA, Aguiar EVD, Capriles VD, Amaral TN, Schmiele M (2024) Use of integral forage palm flour as an innovative ingredient in new fettuccine-type pasta: Thermomechanical and technological properties, and sensory acceptance. Foods 13:2683. <https://doi.org/10.3390/foods13172683>

5. Anushree RK, Kushwaha A, Panwar RK et al (2025) Effect of lentil flour incorporation on the sensory, nutritional, and functional properties of noodles. Discov Food 5:390. <https://doi.org/10.1007/s44187-025-00669-z>

6. Pico J, Pismag RY, Laudouze M, Martinez MM (2020) Systematic evaluation of the Folin–Ciocalteu and Fast Blue BB reactions during the analysis of total phenolics in legumes, nuts and plant seeds. Food Funct 11:9868–9880. <https://doi.org/10.1039/D0FO01857K>

7. Paucar-Menacho LM, Martínez-Villaluenga C, Dueñas M, Frias J, Peñas E (2018) Response surface optimisation of germination conditions to improve the accumulation of bioactive compounds and the antioxidant activity in quinoa. Int J Food Sci Technol 53:516–524. <https://doi.org/10.1111/ijfs.13623>

8. Hough G, Wakeling I, Mucci A, Chambers IV E, Gallardo IM, Alves LR (2006) Number of consumers necessary for sensory acceptability tests. Food Qual Prefer 17:522–526. <https://doi.org/10.1016/j.foodqual.2005.07.002>

9. Derringer G, Suich R (1980) Simultaneous optimization of several response variables. J Qual Technol 12:214–219. <https://doi.org/10.1080/00224065.1980.11980968>

10. Noma S, Hayakawa K, Abe C et al (2019) Contribution of α-gliadin alleles to the extensibility of flour dough in Japanese wheat cultivars. J Cereal Sci 86:15–21. <https://doi.org/10.1016/j.jcs.2018.12.017>

11. Zhu F, Li J (2019) Physicochemical and sensory properties of fresh noodles fortified with ground linseed (Linum usitatissimum). LWT 101:847–853. <https://doi.org/10.1016/j.lwt.2018.12.003>

12. Teterycz D, Sobota A, Zarzycki P, Latoch A (2020) Legume flour as a natural colouring component in pasta production. J Food Sci Technol 57:301–309. <https://doi.org/10.1007/s13197-019-04061-5>

**Tables**

**Table S1.** Physicochemical properties, antioxidant-related parameters, instrumental color, and particle size distribution of the raw materials used for pasta preparation

| **Properties** | **Wheat flour (WF)** | **Flaxseed flour (FF)** | **Lupin flour (LF)** |
| --- | --- | --- | --- |
| Moisture (g/100 g) | 8.12 ± 0.02 | 8.52 ± 0.54 | 12.62 ± 0.63 |
| Fat (g/100 g d.w.) | 0.98 ± 0.09 | 35.29 ± 0.03 | 12.57 ± 0.13 |
| Ash (g/100 g d.w.) | 0.57 ± 0.07 | 2.30 ± 0.05 | 3.80 ± 0.15 |
| Protein (g/100 g d.w.) | 13.54 ± 0.03 | 20.77 ± 0.03 | 38.55 ± 0.45 |
| Total dietary fiber (g/100 g d.w.) | 9.25 ± 0.78 | 26.67 ± 0.35 | 32.01 ± 0.83 |
| Digestible carbohydrates (g/100 g d.w.) | 75.66 ± 0.69 | 14.97 ± 0.45 | 13.07 ± 0.64 |
| TSPC (mg GAE/g d.w.) | 55.40 ± 5.56 | 195.80 ± 11.03 | 129.44 ± 10.47 |
| ORAC (µmol TE/g d.w.) | 17.40 ± 1.94 | 7.33 ± 0.58 | 122.68 ± 19.54 |
| **Color** |  |  |  |
| *L** | 89.56 ± 0.22 | 64.99 ± 0.43 | 74.89 ± 0.04 |
| *a** | −0.42 ± 0.01 | 4.26 ± 0.04 | 0.02 ± 0.10 |
| *b** | 10.05 ± 0.10 | 19.71 ± 0.11 | 24.40 ± 0.52 |
| **Particle size (% retained on each sieve)** |  |  |  |
| 30 mesh (600 µm) | 0.49 ± 0.01 | 8.70 ± 0.01 | 9.28 ± 0.01 |
| 60 mesh (250 µm) | 13.66 ± 0.01 | 18.80 ± 0.01 | 83.51 ± 0.02 |
| 80 mesh (180 µm) | 39.08 ± 0.04 | 62.50 ± 0.04 | 5.09 ± 0.03 |
| 120 mesh (125 µm) | 30.81 ± 0.02 | 7.90 ± 0.02 | 1.82 ± 0.01 |
| Bottom (<125 µm) | 15.95 ± 0.01 | 2.10 ± 0.01 | 0.31 ± 0.02 |

Data are expressed as mean ± standard deviation. Particle size values are expressed as the percentage retained on each sieve. WF, wheat flour; FF, flaxseed flour; LF, lupin flour; d.w., dry weight; TSPC, total soluble phenolic compounds; GAE, gallic acid equivalents; ORAC, oxygen radical absorbance capacity; TE, Trolox equivalents; *L**, lightness; *a**, redness/greenness; *b**, yellowness/blueness

**Table S2.** Instrumental color of raw pasta, cooking properties and texture characteristics of pasta formulated with lupin (LF) and flaxseed (FF) flours

| Formulation | Instrumental color | | | Cooking parameters | | | Instrumental Texture | | | | |
| --- | --- | --- | --- | --- | --- | --- | --- | --- | --- | --- | --- |
|  | *L** | *a** | *b** | Cooking  time (min) | Weight gain (%) | Solid loss (%) | Elasticity (N) | Firmness (N) | Work of shear (N.s) | Stickness (N) | Fracturability (N) |
| Control | 81.00 ± 0.02 | 0.89 ± 0.02 | 14.79 ± 0.03 | 6 | 111.06 ± 5.55 | 0.47 ± 0.02 | 1.5 ± 0.09 | 2.68 ± 0.24 | 1.80 ± 0.20 | -0.28 ± 0.04 | 2.57 ± 0.30 |
| F1 | 72.23 ± 0.05 | 0.17 ± 0.03 | 13.44 ± 0.05 | 6 | 99.02 ± 4.95 | 1.29 ± 0.06 | 0.55 ± 0.05 | 2.07 ± 0.19 | 1.18 ± 0.09 | -0.36 ± 0.04 | 1.91 ± 0.22 |
| F2 | 67.90 ± 0.03 | 0.21 ± 0.11 | 14.31 ± 0.04 | 7 | 145.36 ± 7.26 | 1.36 ± 0.07 | 0.47 ± 0.06 | 1.88 ± 0.12 | 1.06 ± 0.11 | -0.18 ± 0.02 | 1.67 ± 0.17 |
| F3 | 67.32 ± 0.07 | 0.22 ± 0.04 | 12.75 ± 0.04 | 8 | 120.19 ± 6.01 | 1.05 ± 0.05 | 0.36 ± 0.07 | 1.89 ± 0.16 | 1.11 ± 0.11 | -0.15 ± 0.04 | 1.36 ± 0.14 |
| F4 | 44.56 ± 0.07 | 22.48 ± 0.02 | 28.41 ± 0.03 | 8 | 115.53 ± 5.77 | 1.04 ± 0.05 | 0.41 ± 0.05 | 1.73 ± 0.15 | 1.08 ± 0.09 | -0.22 ± 0.03 | 1.14 ± 0.17 |
| F5 | 68.62 ± 0.07 | 0.74 ± 0.08 | 10.12 ± 0.05 | 7 | 114.43 ± 5.72 | 1.18 ± 0.06 | 0.67 ± 0.03 | 2.12 ± 0.27 | 1.03 ± 0.11 | -0.25 ± 0.05 | 1.61 ± 0.15 |
| F6 | 57.77 ± 0.04 | 16.56 ± 0.03 | 37.28 ± 0.1 | 7 | 142.80 ± 7.14 | 1.15 ± 0.06 | 0.55 ± 0.05 | 2.21 ± 0.17 | 1.22 ± 0.14 | -0.44 ± 0.08 | 1.56 ± 0.18 |
| F7 | 64.97 ± 0.05 | 4.31 ± 0.05 | 41.99 ± 0.07 | 6 | 128.36 ± 6.41 | 1.49 ± 0.07 | 0.48 ± 0.04 | 2.28 ± 0.26 | 1.27 ± 0.17 | -0.29 ± 0.03 | 1.45 ± 0.12 |
| F8 | 41.59 ± 0.03 | 8.80 ± 0.04 | 21.08 ± 0.07 | 7 | 109.09 ± 5.45 | 1.29 ± 0.06 | 0.42 ± 0.05 | 2.05 ± 0.18 | 1.23 ± 0.09 | -0.18 ± 0.04 | 1.18 ± 0.08 |
| F9 | 51.61 ± 0.08 | 6.96 ± 0.04 | 22.67 ± 0.04 | 8 | 93.53 ± 4.67 | 1.35 ± 0.07 | 0.4 ± 0.05 | 2.09 ± 0.13 | 1.16 ± 0.13 | -0.19 ± 0.04 | 1.06 ± 0.14 |
| F10 | 51.40 ± 0.02 | 6.84 ± 0.07 | 21.82 ± 0.06 | 8 | 94.06 ± 4.70 | 1.37 ± 0.07 | 0.41 ± 0.07 | 2.06 ± 0.24 | 1.16 ± 0.1 | -0.19 ± 0.03 | 1.09 ± 0.13 |
| F11 | 50.89 ± 0.04 | 6.73 ± 0.04 | 22.75 ± 0.05 | 8 | 94.53 ± 4.73 | 1.35 ± 0.07 | 0.41 ± 0.06 | 2.07 ± 0.12 | 1.16 ± 0.12 | -0.19 ± 0.03 | 1.07 ± 0.14 |
| *P*-value | 0.004 | 0.007 | 0.486 | 0.042 | <0.001 | <0.001 | 0.044 | 0.511 | 0.299 | 0.013 | 0.417 |
| R^2^ | 0.9429 | 0.9259 | 0.5084 | 0.8466 | 0.9791 | 0.8536 | 0.7627 | 0.4935 | 0.6218 | 0.9073 | 0.5493 |

**Table S3.** Total soluble phenolic compounds (TSPC) and antioxidant capacity (ORAC) of pasta formulated with lupin (LF) and flaxseed (FF) flours in dry weight (d.w).

| Formulations | TSPC  (mg GAE/100 g d.w.) | ORAC  (µmol TE/g d.w.) |
| --- | --- | --- |
| Control | 121.03 ± 1.94 | 15.46 ± 1.11 |
| F1 | 289.85 ± 5.18 | 35.99 ± 1.01 |
| F2 | 310.54 ± 4.46 | 49.72 ± 0.05 |
| F3 | 348.99 ± 5.62 | 55.62 ± 0.90 |
| F4 | 297.85 ± 5.42 | 63.12 ± 1.11 |
| F5 | 282.20 ± 4.05 | 49.55 ± 0.12 |
| F6 | 301.03 ± 3.65 | 49.11± 0.64 |
| F7 | 276.85 ± 2.30 | 45.3 ± 0.44 |
| F8 | 307.69 ± 2.57 | 59.54 ± 1.57 |
| F9 | 274.70 ± 3.98 | 51.39 ± 1.87 |
| F10 | 272.70 ± 10.31 | 51.36 ± 1.10 |
| F11 | 274.70 ± 8.44 | 51.35 ± 0.10 |
| *P*-value | 0.104 | 0.045 |
| R^2^ | 0.7716 | 0.8417 |

Data represent the mean ± standard deviation of three replicates; TSPC = total soluble phenolic content; ORAC= oxygen radical absorbance capacity GAE = gallic acid equivalent; TE = trolox equivalent.

**Table S4.** Acceptance scores of functional pastas formulated with lupin (LF) and flaxseed (FF) flours

| Formulation | Sensory acceptance | | | | Purchase intention |
| --- | --- | --- | --- | --- | --- |
|  | Color | Flavor | Texture | Appearance |  |
| Control | 7.58 ± 0.87 | 7.60 ± 1.53 | 7.63 ± 1.21 | 7.45 ± 1.13 | 4.23 ± 1.10 |
| F1 | 7.08 ± 1.19 | 6.65 ± 1.55 | 7.23 ± 1.17 | 6.30 ± 1.26 | 3.65 ± 0.92 |
| F2 | 6.10 ± 1.52 | 6.83 ± 1.50 | 6.95 ± 1.38 | 6.50 ± 1.45 | 3.75 ± 0.90 |
| F3 | 6.43 ± 1.57 | 6.25 ± 1.69 | 6.68 ± 1.46 | 6.48 ± 1.68 | 3.58 ± 1.22 |
| F4 | 6.75 ± 1.58 | 6.10 ± 1.66 | 6.38 ± 1.50 | 6.75 ± 1.72 | 3.28 ± 0.96 |
| F5 | 6.95 ± 1.34 | 6.65 ± 1.37 | 6.75 ± 1.24 | 7.00 ± 1.32 | 3.88 ± 1.14 |
| F6 | 6.83 ± 1.57 | 6.88 ± 1.52 | 7.05 ± 1.24 | 6.78 ± 1.53 | 3.90 ± 0.90 |
| F7 | 7.60 ± 1.13 | 6.85 ± 1.08 | 7.35 ± 0.92 | 7.25 ± 1.15 | 4.15 ± 0.74 |
| F8 | 5.90 ± 1.81 | 6.75 ± 1.39 | 6.80 ± 1.22 | 6.83 ± 1.28 | 3.68 ± 1.10 |
| F9 | 6.53 ± 1.89 | 6.78 ± 1.51 | 6.65 ± 1.49 | 6.25 ± 1.72 | 3.83 ± 0.96 |
| F10 | 6.73 ± 1.26 | 6.30 ± 1.36 | 6.30 ± 1.64 | 6.30 ± 1.36 | 3.15 ± 0.92 |
| F11 | 6.68 ± 1.25 | 6.38 ± 1.25 | 6.75 ± 1.45 | 6.85 ± 1.51 | 3.75 ± 0.95 |
| *P*-value | 0.354 | 0.708 | 0.195 | 0.941 | 0.735 |
| R^2^ | 0.5875 | 0.3734 | 0.6943 | 0.1780 | 0.3558 |

**Figures**

**Figure S1.** Fresh pasta formulations obtained with different levels of lupin flour (LF) and flaxseed flour (FF) substitution, and a control with wheat flour (WF)

**Figure S2.** Scatter plot showing the Pearson correlation between sensory texture scores and instrumental firmness (N) of pasta samples.

**Figure S3.** Pearson correlation between sensory texture and fracturability (N) of pasta samples.

**Figure S4.** Pearson correlation between overall acceptability (IA) and global sensory score of pasta samples.

**Figure S5.** Pearson correlation between overall acceptability and instrumental stickiness (N) of pasta samples.

**Figure S6.** Pearson correlation between overall acceptability and cooking time (min) of pasta samples.

**Figure S7.** Pearson correlation between overall acceptability and whole linseed flour (%) in pasta formulations.

**Figure S8.** Pearson correlation between overall acceptability (IA) and purchase intention (IC) of pasta samples.

**Figure S9.** Pearson correlation between overall acceptability (IA) and sensory texture of pasta samples.

**Figure S10.** Pearson correlation between overall acceptability (IA) and cooking time (min) of pasta samples.

**Figure S11.** Pearson correlation between overall acceptability (IA) and whole linseed flour (%) in pasta formulations.

**Figure S12.** Pearson correlation between sensory flavor and cooking time (min) of pasta samples.

**Figure S13.** Pearson correlation between sensory flavor and instrumental stickiness (N) of pasta samples.

**Figure S14.** Pearson correlation between sensory texture and whole linseed flour (%) in pasta formulations.

**Figure S15.** Pearson correlation between sensory texture and cooking time (min) of pasta samples.

**Figure S16.** Numerical optimization plot showing the effects of whole lupin flour and whole flaxseed flour on technological, sensory-related, and antioxidant responses of pasta formulations.
